# Supplementary material for: BRCA2 abrogation triggers innate immune responses potentiated by treatment with PARP inhibitors
Source: Nat Commun. 2019 Jul 17;10:3143. doi: 10.1038/s41467-019-11048-5 (PMC6637138; doi:10.1038/s41467-019-11048-5)
Supplement: Supplementary file 1 — Supplementary Information [file 41467_2019_11048_MOESM1_ESM.pdf]

Supplementary Information

BRCA2 abrogation triggers innate immune responses potentiated by treatment with PARP inhibitors

Reisländer *et al.*

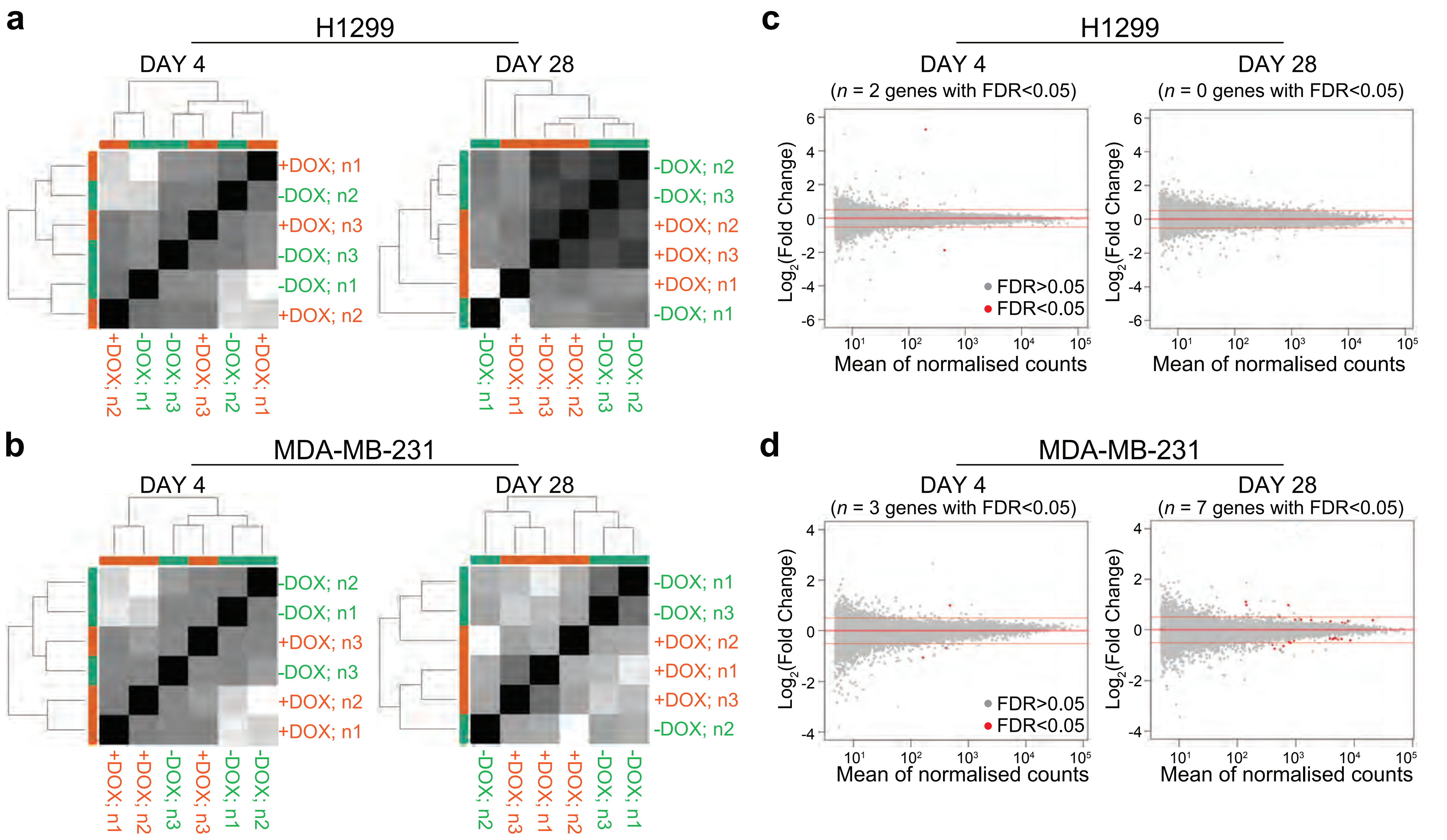

Supplementary Figure 1. Doxycycline (DOX) treatment does not induce transcriptional changes in parental H1299 or MDA-MB-231 human cells. (**a**, **b**) Euclidean sample-to-sample distances calculated from RNA-seq normalised count data for H1299 (**a**) and MDA-MB-231 (**b**) cells treated with 2 µg/ml DOX for 4 or 28 days. Hierarchical clustering of distance matrices shows no clear distinction between +DOX and -DOX samples. (**c**, **d**) MA plots showing differentially expressed genes between +DOX and -DOX samples treated as in (**a**, **b**). Each dot represents one gene; red, genes with significantly altered expresssion values (FDR < 0.05); gray, genes without significantly altered expresssion values (FDR > 0.05).

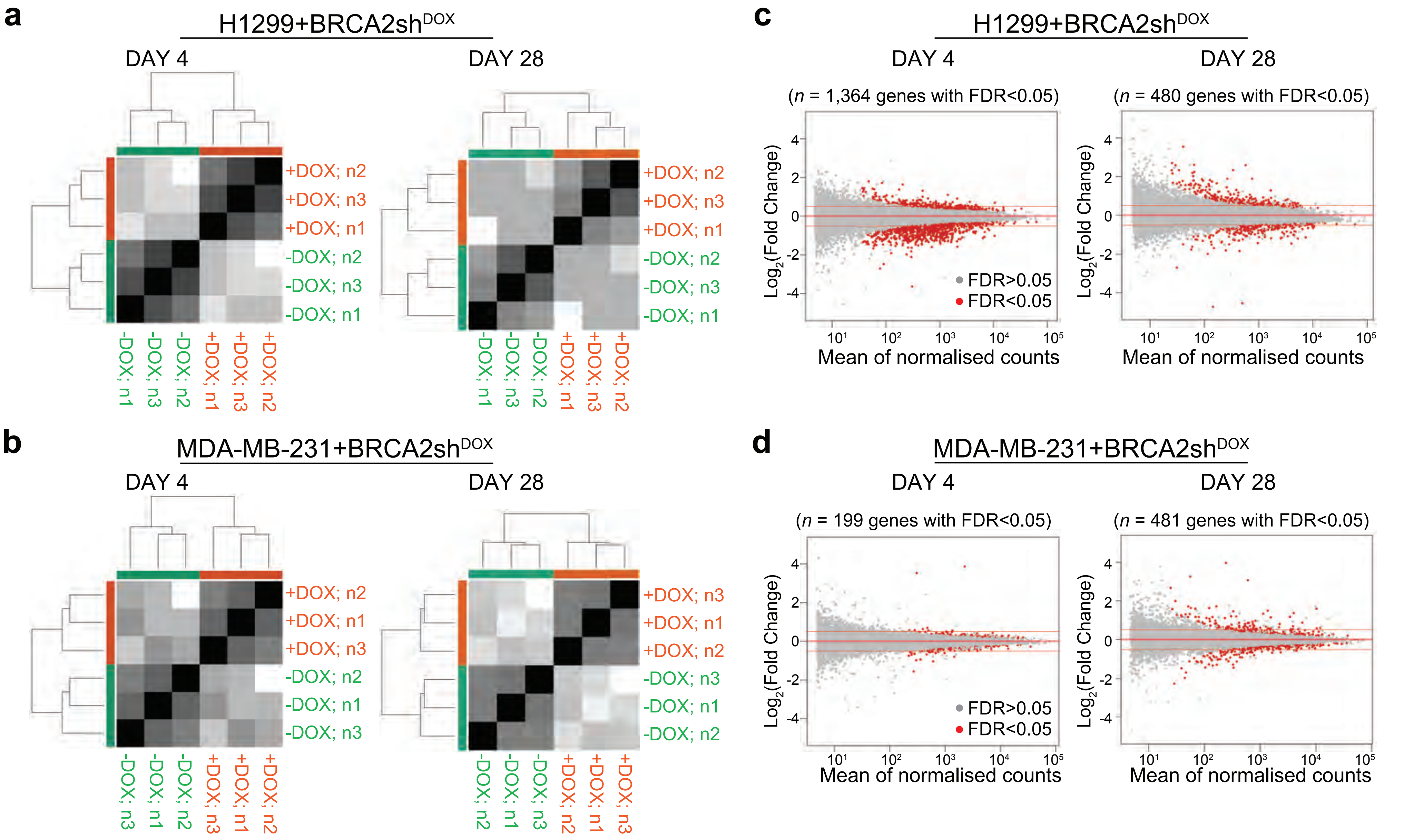

Supplementary Figure 2. Doxycycline (DOX) treatment induces significant transcriptional changes in H1299 or MDA-MB-231 human cells expressing a DOX-inducible shRNA against *BRCA2*. (**a**, **b**) Euclidean sample-to-sample distances calculated from RNA-seq normalized count data for H1299 (**a**) and MDA-MB-231 (**b**) cells carrying a DOX-inducible shRNA against *BRCA2*, treated with 2 µg/ml DOX for 4 or 28 days. Hierarchical clustering of distance matrices shows a clear distinction between -DOX and +DOX samples. (**c**, **d**) MA plots showing differentially expressed genes between +DOX and -DOX samples treated as in (**a**, **b**). Each dot represents one gene; red, genes with significantly altered expresssion values (FDR < 0.05); gray, genes without significantly altered expresssion values (FDR > 0.05).

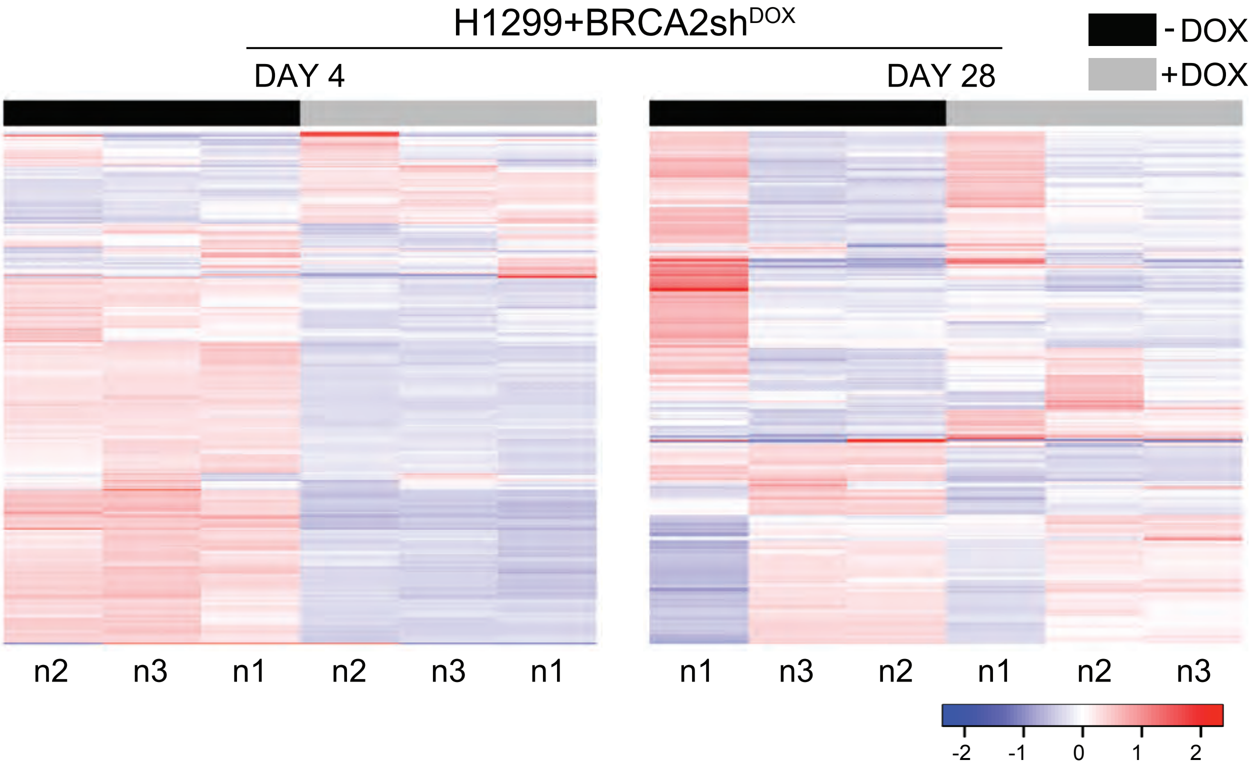

Supplementary Figure 3. Supervised clustering of log-transformed gene counts. Data were obtained from *n* = 3 independent RNA-seq experiments performed in H1299 cells expressing a DOX-inducible shRNA against BRCA2. Top scoring 500 genes are shown.

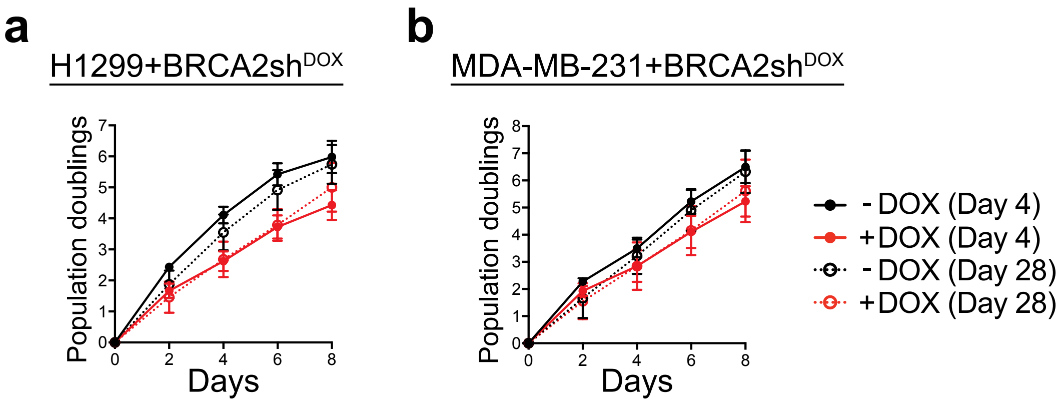

Supplementary Figure 4. Proliferation capacity of H1299 and MDA-MB-231 cells upon short-term (4 days) or long-term (28 days) BRCA2 inactivation using a DOX-inducible shRNA. Viability of H1299 (**a**) and MDA-MB-231 (**b**) cells was determined over a period of 8 days and reported to day 0 in order to calculate population doublings. Day 0 represents either day 4 (solid line) or day 28 (dotted line) after DOX addition. Error bars represent SD of *n* = 3 independent experiments.

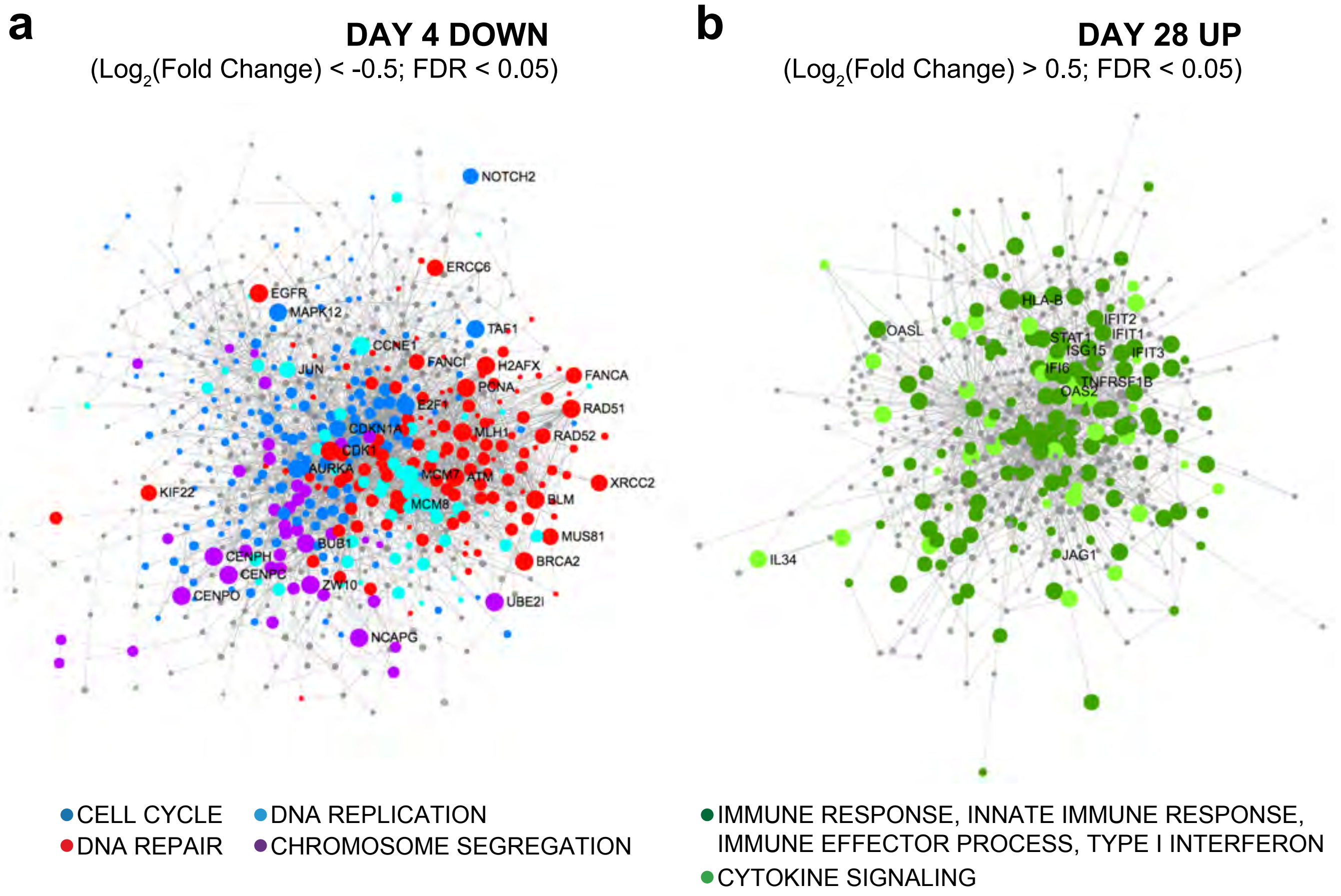

Supplementary Figure 5. Protein-protein interaction networks in H1299 cells during short-term (4 days) or long-term (28 days) BRCA2 inactivation using a DOX-inducible shRNA. First-order protein-protein interaction networks between genes downregulated after 4 days (**a**) or upregulated after 28 days (**b**) of DOX treatment. Node size is proportional to the number of its connections with other nodes. n = 574 downregulated genes (day 4) and n = 213 upregulated genes (day 28) were used for network analysis, resulting in 669 and 446 nodes, respectively.

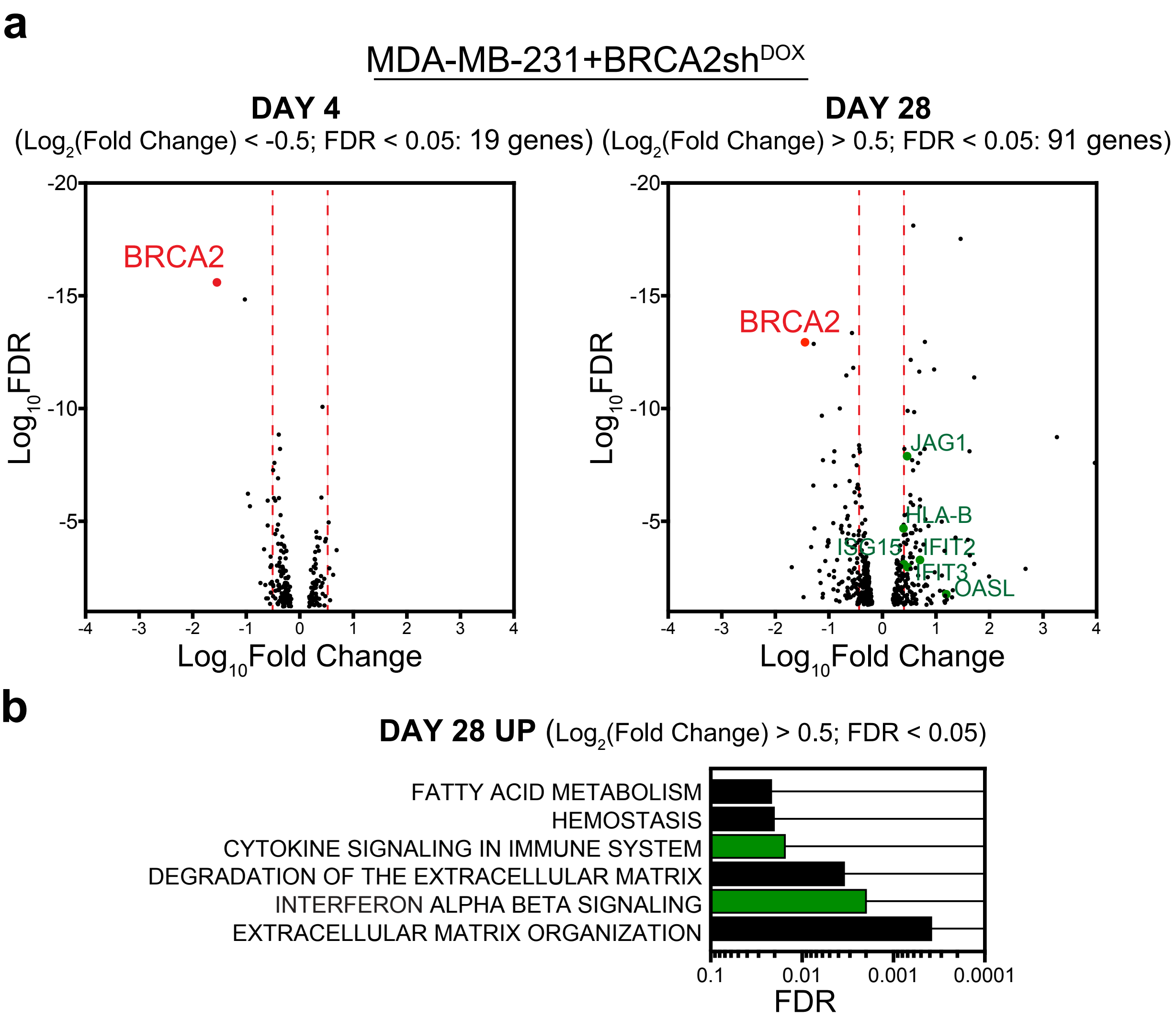

Supplementary Figure 6. Differential gene expression analyses of MDA-MB-231 cells carrying a DOX-inducible shRNA targeting BRCA2. (**a**) Volcano plot of genes differentially expressed (FDR < 0.05) in BRCA2-deficient (+DOX) versus BRCA2-proficient (-DOX) cells, assessed at day 4 or day 28 after DOX addition. (**b**) REACTOME pathway-based enrichment analysis of genes upregulated (Log$_2$(Fold Change) > 0.5; FDR < 0.05) in +DOX relative to -DOX samples following 28 days of DOX treatment.

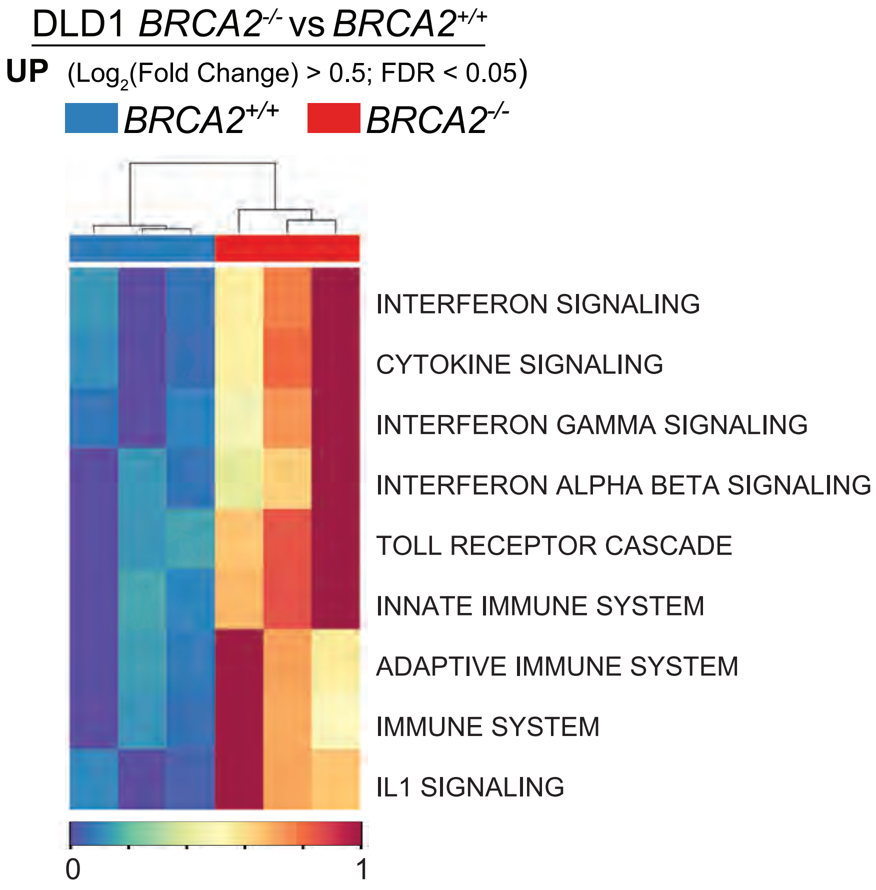

Supplementary Figure 7. Pathway deregulation score analysis for genes significantly upregulated (Log_2(Fold Change) > 0.5; FDR < 0.05) in BRCA2$^{-/-}$ vs BRCA2$^{+/+}$ DLD1 cells. Each row corresponds to a Reactome pathway and each column to an RNA-seq sample.

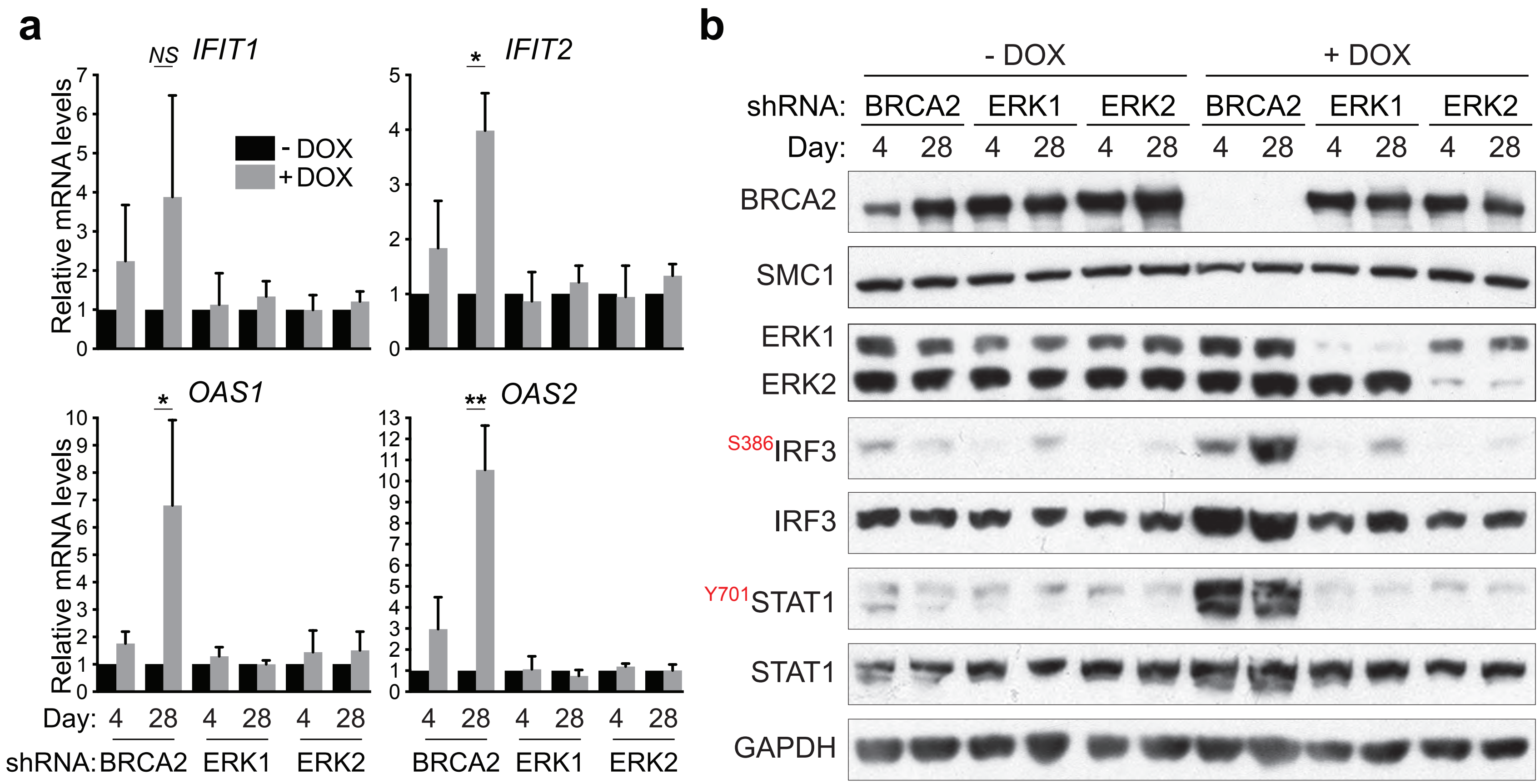

Supplementary Figure 8. Upregulation of innate immune response genes is not triggered by ERK1 or ERK2 inactivation. H1299 cells expressing a doxycycline (DOX)-inducible BRCA2 shRNA were grown in the presence or absence of DOX for 28 days. Cells were subjected to (**a**) quantitative RT-PCR analyses using primers specific for the indicated genes. mRNA levels were expressed relative to the gene encoding β-actin and to untreated (-DOX) controls ($2^{-\Delta\Delta CT}$). Error bars represent SD of $n = 3$ independent experiments, each performed in triplicates. *NS*, $p > 0.05$; *, $p < 0.05$; **, $p < 0.01$ (unpaired two-tailed t test). (**b**) Whole cell extracts were immunoblotted as indicated. GAPDH and SMC1 were used as loading control. Phosphorylation sites are indicated in red.

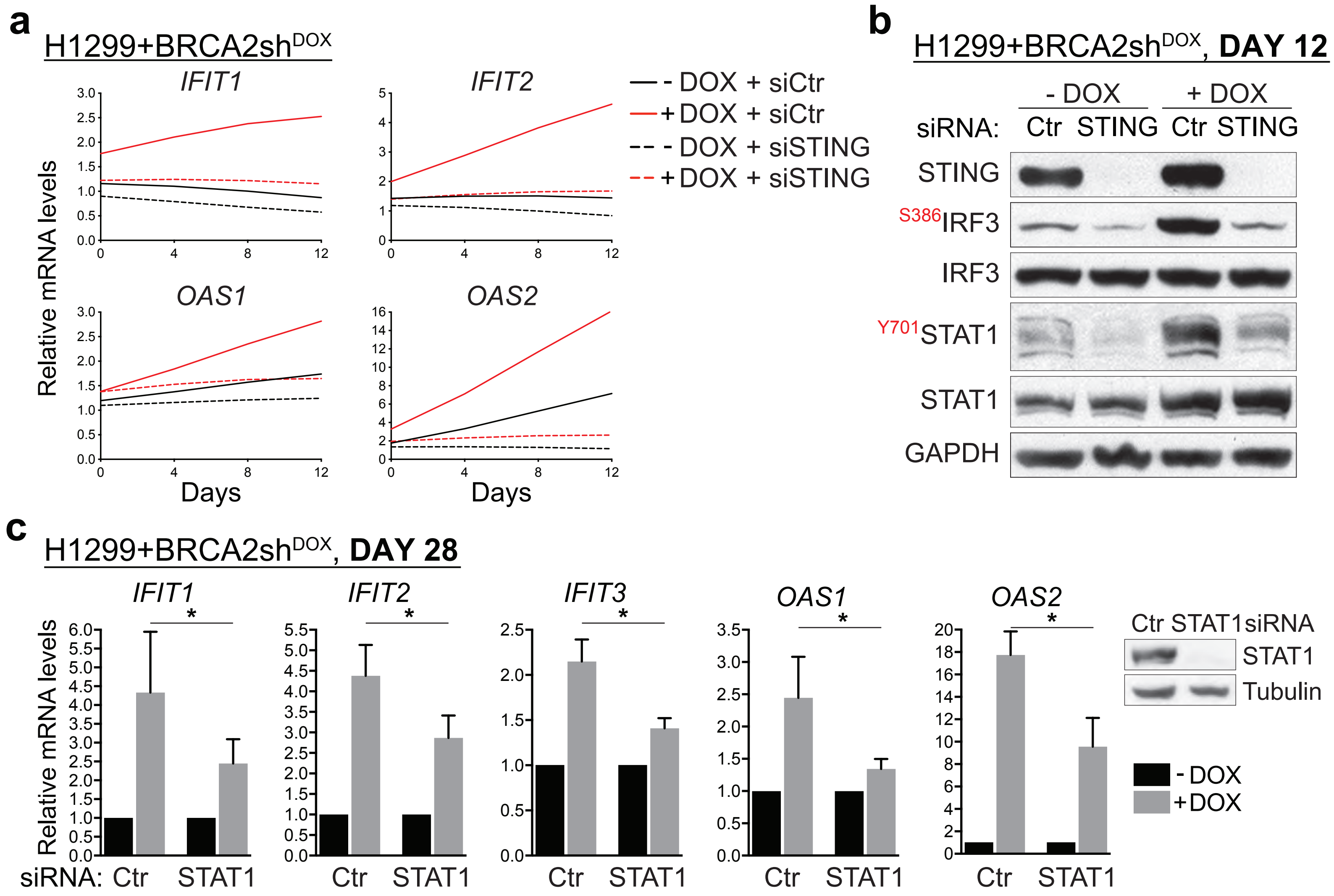

Supplementary Figure 9. Upregulation of the innate immune response genes triggered by BRCA2 inactivation is STING and STAT1-dependent. H1299 cells expressing a doxycycline (DOX)-inducible BRCA2 shRNA were grown in the presence or absence of DOX. (**a**, **b**) Cells transfected with STING siRNA every 4 days were subjected to (**a**) quantitative RT-PCR analyses using primers specific for the indicated genes. mRNA levels were expressed relative to the gene encoding β-actin and to day 0 ($2^{-\Delta\Delta CT}$). (**b**) Whole cell extracts were immunoblotted as indicated. GAPDH was used as a loading control. Phosphorylation sites are indicated in red. (**c**) Upon 28 days of DOX treatment, cells were transfected with STAT1 siRNA for 48 hours and subjected to quantitative RT-PCR analyses using primers specific for the indicated genes. mRNA levels were expressed relative to the gene encoding β-actin and to untreated (-DOX) controls ($2^{-\Delta\Delta CT}$). Error bars represent SD of $n = 4$ independent experiments, each performed in triplicates. *, $p < 0.05$ (unpaired two-tailed t test). Whole cell extracts were immunoblotted as indicated. Tubulin was used as a loading control.

Ovarian serous cystadenocarcinomas (TCGA, n = 316)

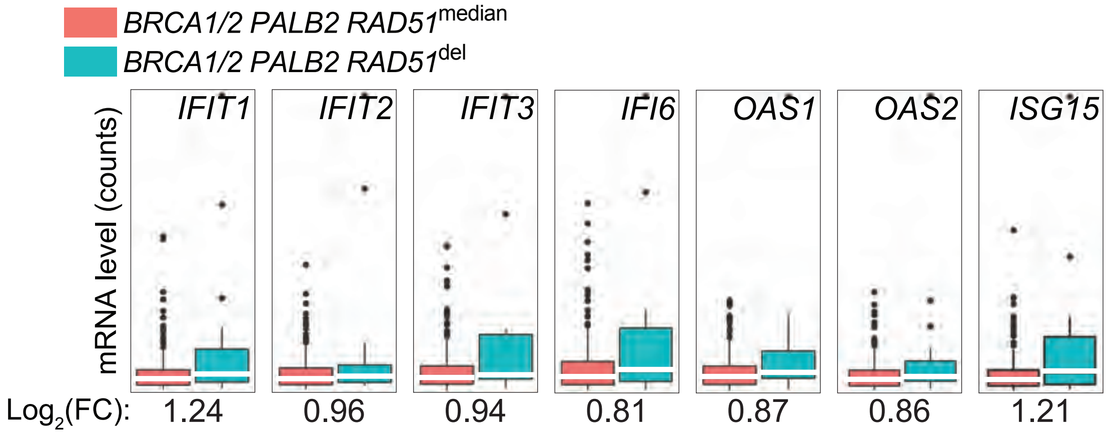

Supplementary Figure 10. Upregulation of innate immune response genes in HR-deficient ovarian tumors.

Upregulation of innate immune response genes in HR-deficient tumors (*BRCA1*-, *BRCA2*-, *PALB2*- or *RAD51*-deleted ovarian tumors; n = 17) versus the group of tumours with median mRNA expression (n = 257 samples). Dots in graphs represent individual tumors. Middle line (white), median; box limits 25 and 75 percentiles; whiskers, minimum and maximum values.
