# Supplementary material for: BRCA2 abrogation triggers innate immune responses potentiated by treatment with PARP inhibitors
Source: Nat Commun. 2019 Jul 17;10:3143. doi: 10.1038/s41467-019-11048-5 (PMC6637138; doi:10.1038/s41467-019-11048-5)
Supplement: Supplementary file 3 — Description of Additional Supplementary Files [file 41467_2019_11048_MOESM3_ESM.pdf]

Description of Additional Supplementary Files

File Name: Supplementary Data 1
Description:

1A: List of genes significantly deregulated (FDR < 0.05) after 4 days of DOX treatment relative to untreated cells, in H1299 human cells expressing a DOX-inducible BRCA2 shRNA
1B: List of genes significantly deregulated (FDR < 0.05) after 4 days of DOX treatment relative to untreated cells, in MDA-MB-231 human cells expressing a DOX-inducible BRCA2 shRNA

File Name: Supplementary Data 2
Description:

2A: List of genes significantly deregulated (FDR < 0.05) after 28 days of DOX treatment relative to untreated cells, in H1299 human cells expressing a DOX-inducible BRCA2 shRNA
2B: List of genes significantly deregulated (FDR < 0.05) after 28 days of DOX treatment relative to untreated cells, in MDA-MB-231 human cells expressing a DOX-inducible BRCA2 shRNA
