# Supplementary material for: BRCA2 abrogation triggers innate immune responses potentiated by treatment with PARP inhibitors
Source: Nat Commun. 2019 Jul 17;10:3143. doi: 10.1038/s41467-019-11048-5 (PMC6637138; doi:10.1038/s41467-019-11048-5)
Supplement: Supplementary file 6 — Reporting Summary [file 41467_2019_11048_MOESM6_ESM.pdf]

nature research

Corresponding author(s): Tarsounas

Last updated by author(s): 06 June 2019

Reporting Summary

Nature Research wishes to improve the reproducibility of the work that we publish. This form provides structure for consistency and transparency in reporting. For further information on Nature Research policies, see Authors & Referees and the Editorial Policy Checklist.

Statistics

For all statistical analyses, confirm that the following items are present in the figure legend, table legend, main text, or Methods section.

n/a	Confirmed	
☐	☒	The exact sample size (*n*) for each experimental group/condition, given as a discrete number and unit of measurement
☐	☒	A statement on whether measurements were taken from distinct samples or whether the same sample was measured repeatedly
☐	☒	The statistical test(s) used AND whether they are one- or two-sided<br>*Only common tests should be described solely by name; describe more complex techniques in the Methods section.*
☒	☐	A description of all covariates tested
☐	☒	A description of any assumptions or corrections, such as tests of normality and adjustment for multiple comparisons
☐	☒	A full description of the statistical parameters including central tendency (e.g. means) or other basic estimates (e.g. regression coefficient) AND variation (e.g. standard deviation) or associated estimates of uncertainty (e.g. confidence intervals)
☐	☒	For null hypothesis testing, the test statistic (e.g. *F*, *t*, *r*) with confidence intervals, effect sizes, degrees of freedom and *P* value noted<br>*Give P values as exact values whenever suitable.*
☒	☐	For Bayesian analysis, information on the choice of priors and Markov chain Monte Carlo settings
☐	☒	For hierarchical and complex designs, identification of the appropriate level for tests and full reporting of outcomes
☒	☐	Estimates of effect sizes (e.g. Cohen's *d*, Pearson's *r*), indicating how they were calculated

Our web collection on statistics for biologists contains articles on many of the points above.

Software and code

Policy information about availability of computer code

Data collection	Omega V5.10 R2, Omega Data Analysis V3.02 R2
Data analysis	MS Excel V16.18, Graphpad Prism V7, FlowJo V9.4

For manuscripts utilizing custom algorithms or software that are central to the research but not yet described in published literature, software must be made available to editors/reviewers. We strongly encourage code deposition in a community repository (e.g. GitHub). See the Nature Research guidelines for submitting code & software for further information.

Data

Policy information about availability of data

All manuscripts must include a data availability statement. This statement should provide the following information, where applicable:

- Accession codes, unique identifiers, or web links for publicly available datasets
- A list of figures that have associated raw data
- A description of any restrictions on data availability

GEO submitted (accession number: GSE123631)

Field-specific reporting

Please select the one below that is the best fit for your research. If you are not sure, read the appropriate sections before making your selection.

☒ Life sciences ☐ Behavioural & social sciences ☐ Ecological, evolutionary & environmental sciences

For a reference copy of the document with all sections, see nature.com/documents/nr-reporting-summary-flat.pdf

Life sciences study design

All studies must disclose on these points even when the disclosure is negative.

Sample size	All experiments were performed as biological replicates with at least three independent replicates as indicated in each figure legend. No power analyses were performed because biological replicates of n=3 were considered to have enough power to find biologically significant effects.
Data exclusions	No data points were excluded
Replication	Biological replicates were used within experiments. Further we used multiple cellular models and patient derived data.
Randomization	We allocated randomly 5 animals per dose group which was the minimum needed to achieve the scientific objectives of the study.
Blinding	Microscopy pictures were blinded before quantification.

Reporting for specific materials, systems and methods

We require information from authors about some types of materials, experimental systems and methods used in many studies. Here, indicate whether each material, system or method listed is relevant to your study. If you are not sure if a list item applies to your research, read the appropriate section before selecting a response.

Materials & experimental systems

n/a	Involved in the study	
☐	☒	Antibodies
☐	☒	Eukaryotic cell lines
☒	☐	Palaeontology
☐	☒	Animals and other organisms
☒	☐	Human research participants
☒	☐	Clinical data

Methods

n/a	Involved in the study	
☒	☐	ChIP-seq
☐	☒	Flow cytometry
☒	☐	MRI-based neuroimaging

Antibodies

Antibodies used	The following antibodies were used for immunoblotting: mouse monoclonal antibodies raised against BRCA2 (1:1,000, OP95, Calbiochem), GAPDH (1:30,000, 6C5, Novus Biologicals); rabbit monoclonal antibody raised against ERK1/2 (1:5,000, 4695, Cell Signaling); rabbit polyclonal antibodies raised against STING (1:1,000, CST13647, Cell Signaling), IRF3 (1:1,000, AB76409, Abcam), phospho-IRF3 (1:1,000, AB76493, Abcam), STAT1 (1:1,000, 9175, Cell Signaling), phospho-STAT1 (1:1,000, 9167, Cell Signaling), phospho-KAP1 (1:1,000, A300-767A, Bethyl Laboratories), KAP1 (1:5,000, A300-274A, Bethyl Laboratories), SMC1 (1:5,000, A300-055A, Bethyl Laboratories).<br>The following antibody was used for IF: rabbit monoclonal antibody against cGAS (1:200, 15102, Cell Signaling)
Validation	Validation statements for these antibodies for use with human cells can be found on the manufacturer's websites.

Eukaryotic cell lines

Policy information about cell lines

Cell line source(s)	Human non-small cell lung carcinoma H1299 cells (ATCC) and human invasive ductal breast cancer MDA-MB-231 cells (ATCC) and Human colorectal adenocarcinoma DLD1 cells, parental and BRCA2-mutated (Horizon)
Authentication	Cell lines were authenticated using STR DNA profiling
Mycoplasma contamination	Cells were tested negative for mycoplasma contamination
Commonly misidentified lines<br>(See ICLAC register)	The used cell lines are not listed in the ICLAC database.

Animals and other organisms

Policy information about studies involving animals; ARRIVE guidelines recommended for reporting animal research

Laboratory animals	CB17/SCID mice female (6 weeks old and weighing 26-28 g) were purchased from Charles River Laboratories (Calco, Italy).

Wild animals	N/A
Field-collected samples	N/A
Ethics oversight	All animal procedures were in compliance with the national and international directives (D.L. March 4, 2014, no. 26; directive 2010/63/EU of the European Parliament and of the council; Guide for the Care and Use of Laboratory Animals, United States National Research Council, 2011).

Note that full information on the approval of the study protocol must also be provided in the manuscript.

Flow Cytometry

Plots

Confirm that:

☐ The axis labels state the marker and fluorochrome used (e.g. CD4-FITC).

☐ The axis scales are clearly visible. Include numbers along axes only for bottom left plot of group (a 'group' is an analysis of identical markers).

☐ All plots are contour plots with outliers or pseudocolor plots.

☐ A numerical value for number of cells or percentage (with statistics) is provided.

Methodology

Sample preparation	To label replicated DNA, cells were incubated with 25 μM EdU for 30 min. Samples were collected by trypsinization and fixed using 90% methanol. Incorporated EdU was detected using the Click-iT EdU Alexa Fluor 647 Flow Cytometry Assay Kit (C10634, Invitrogen) according to manufacturer's instructions. Cells were re-suspended in PBS containing 20 μg/mL propidium iodide (P4864, Sigma) and 400 μg/mL RNase A (12091-021, Invitrogen).
Instrument	Samples were processed using flow cytometry (342975, BD FACSCalibur, BD Biosciences).
Software	Cell Quest Pro was used to acquire data. In the end, 10,000 events were analyzed per condition using FlowJo software.
Cell population abundance	N/A
Gating strategy	Forward scatter and side scatter were used to gate for live and single cells. PI signal was used to gate for 2N and 4N populations. EdU signal was used to gate for cells in S-phase.

☐ Tick this box to confirm that a figure exemplifying the gating strategy is provided in the Supplementary Information.
