# Supplementary figures and images for: BRCA2 abrogation triggers innate immune responses potentiated by treatment with PARP inhibitors
Source: Nat Commun. 2019 Jul 17;10:3143. doi: 10.1038/s41467-019-11048-5 (PMC6637138; doi:10.1038/s41467-019-11048-5)

**b**

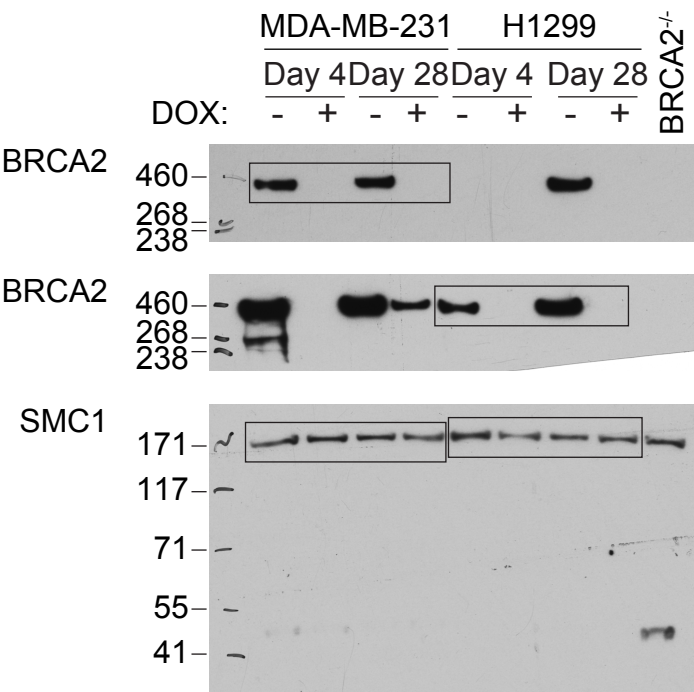

C

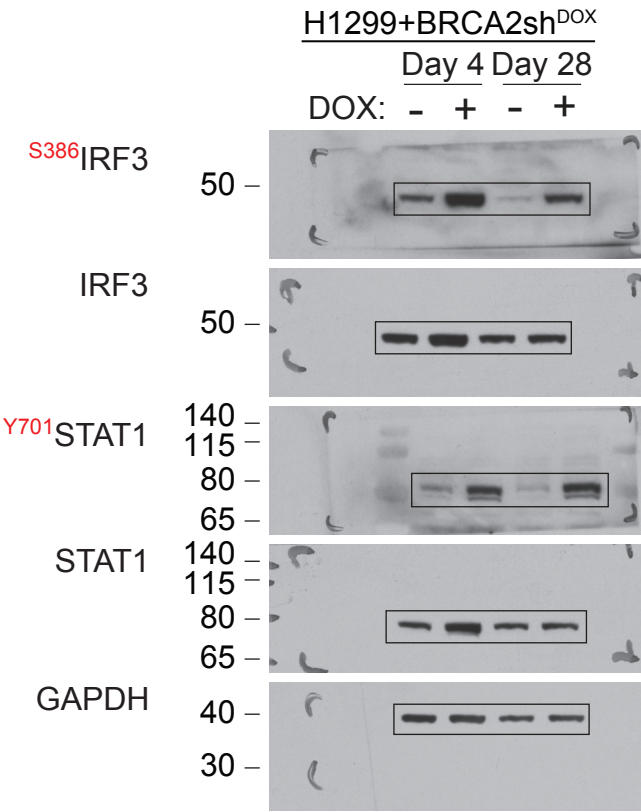

d

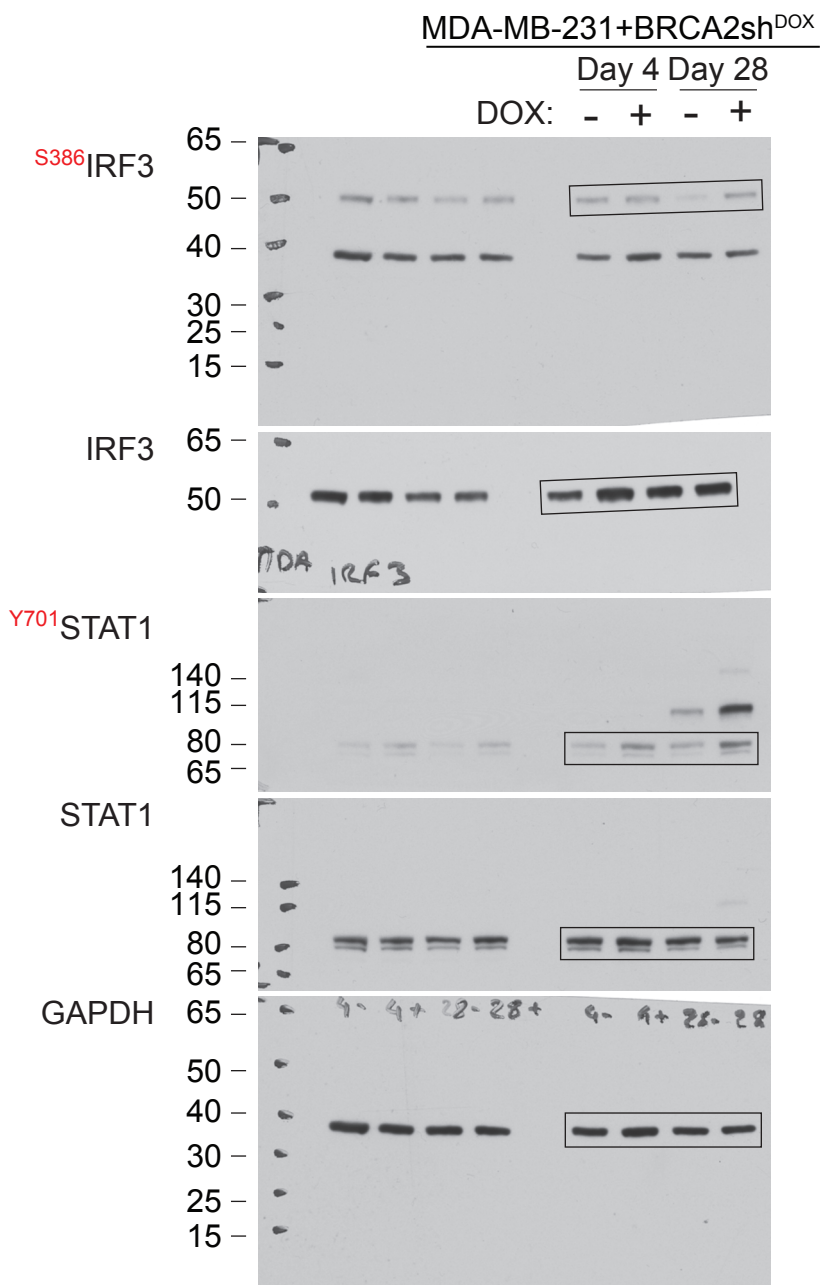

**b**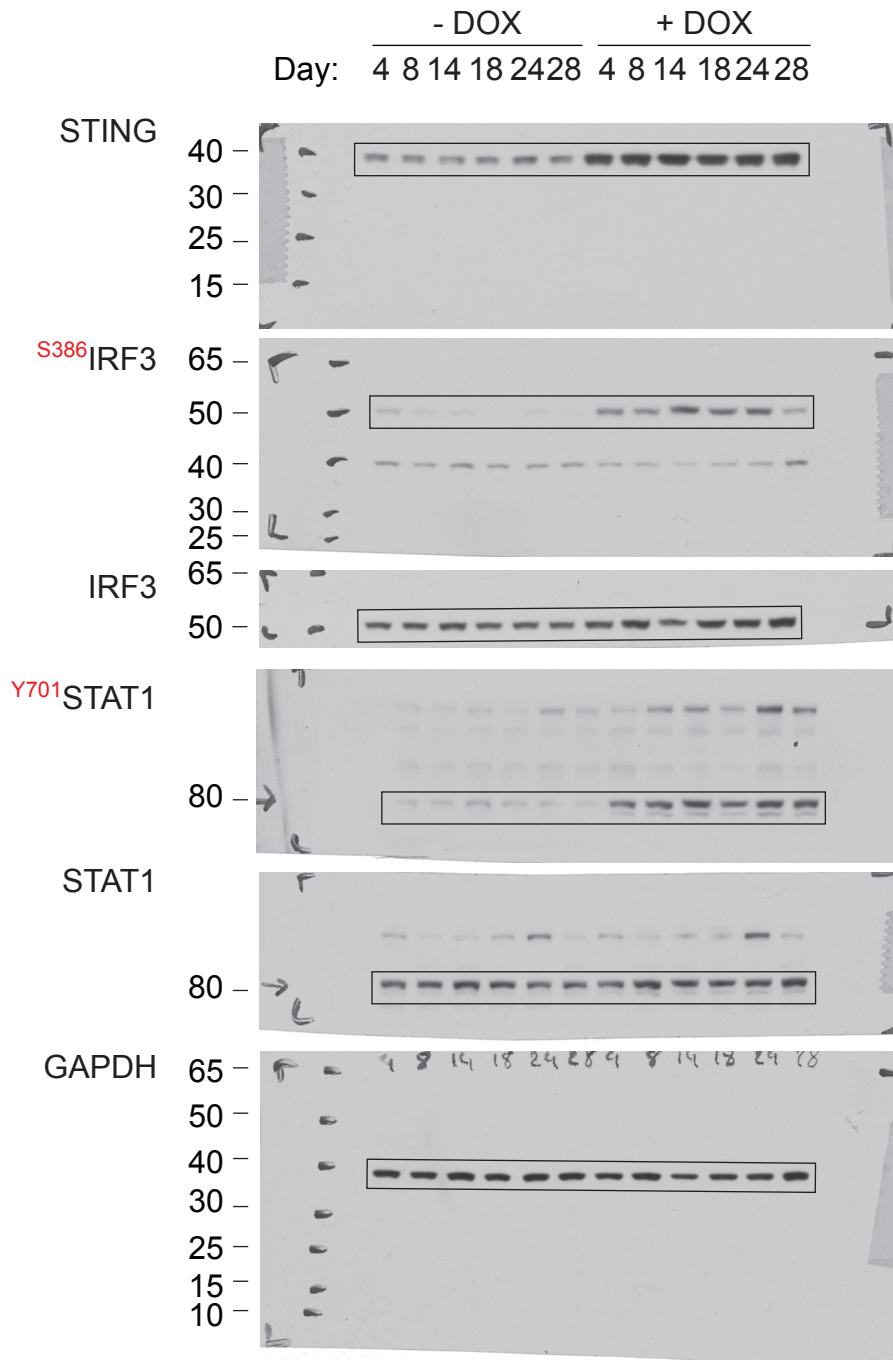

**b**

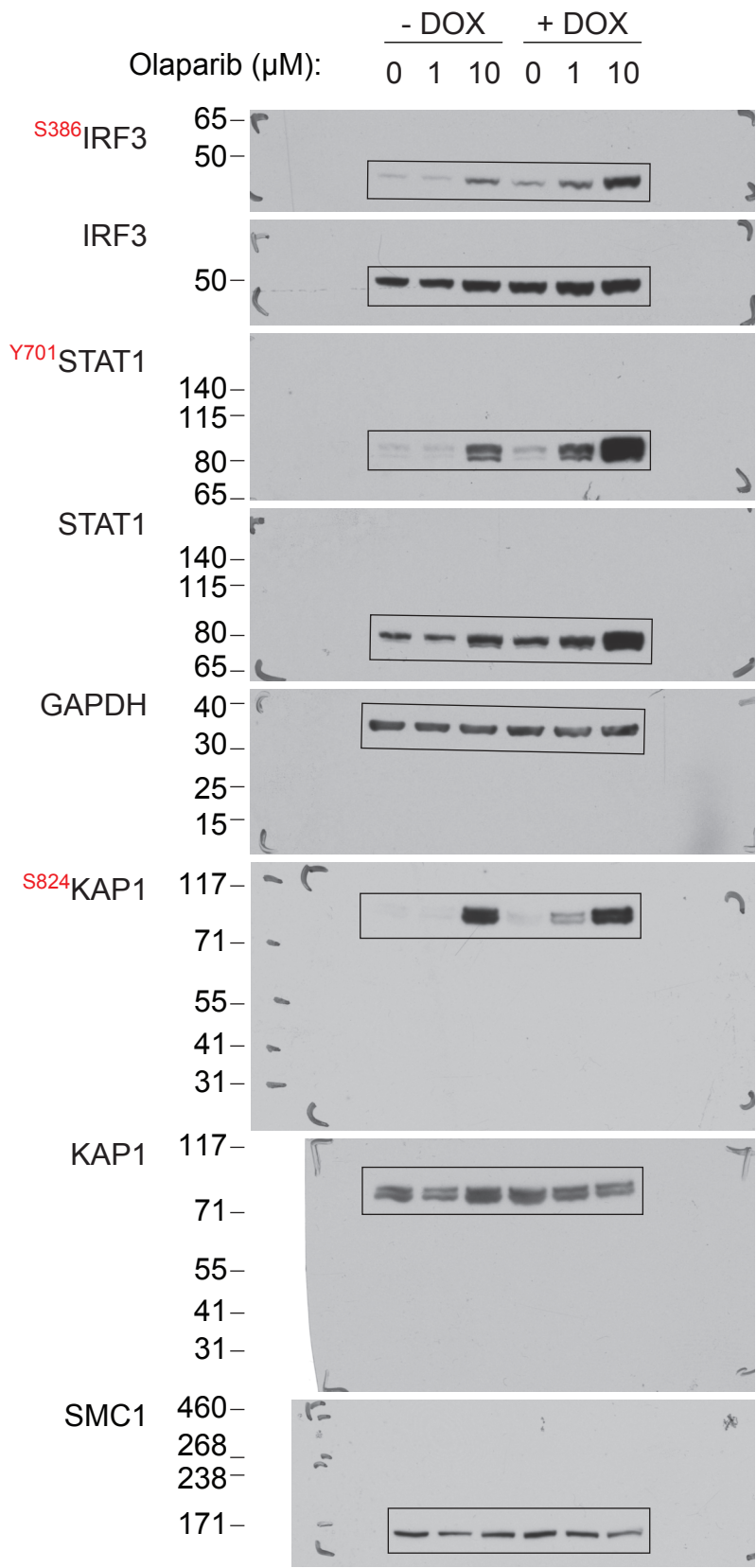

**b**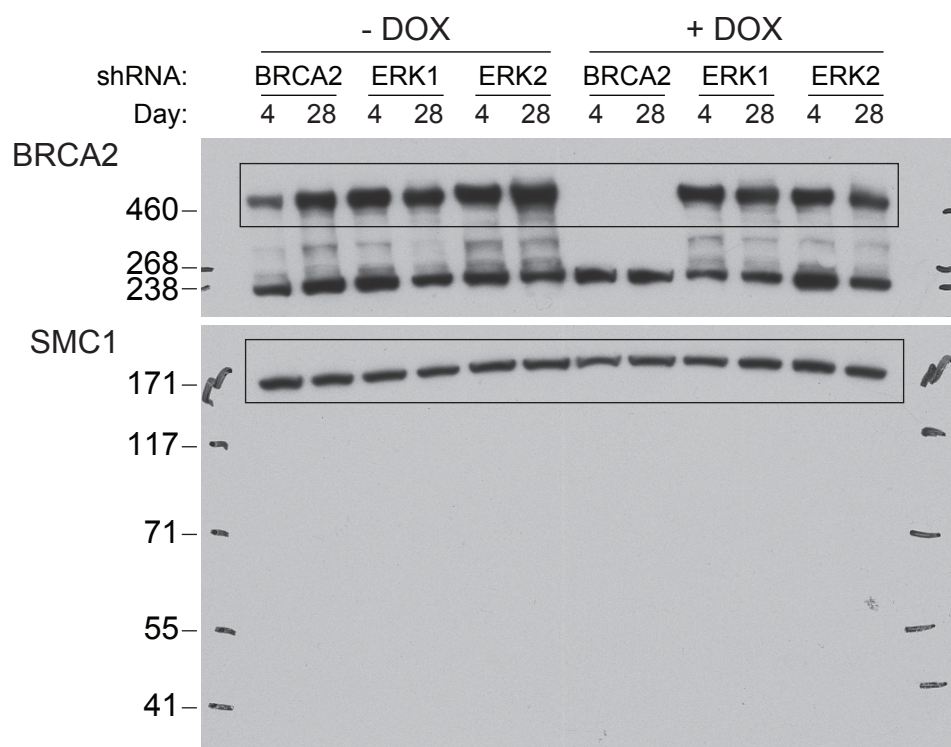

**b**

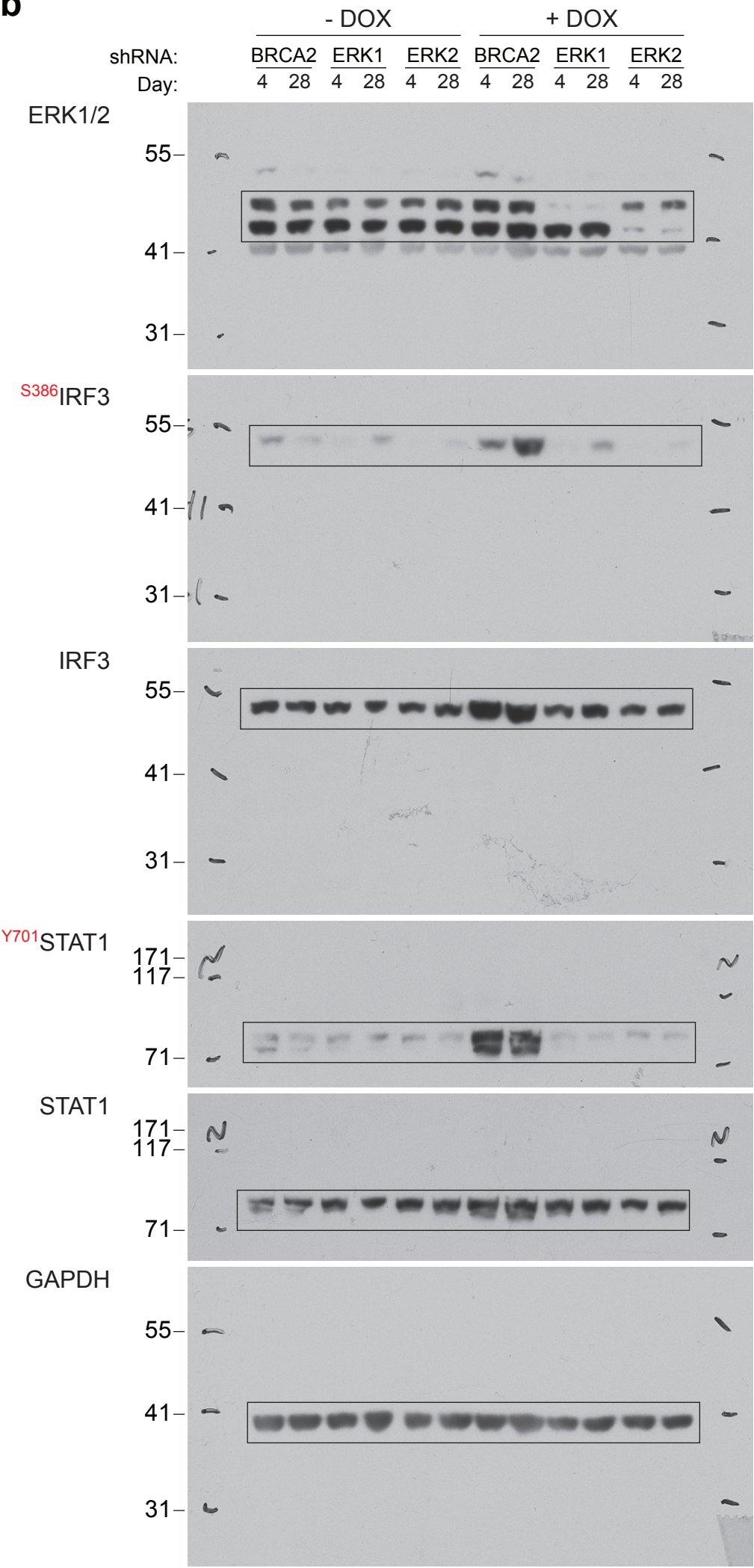

**b**

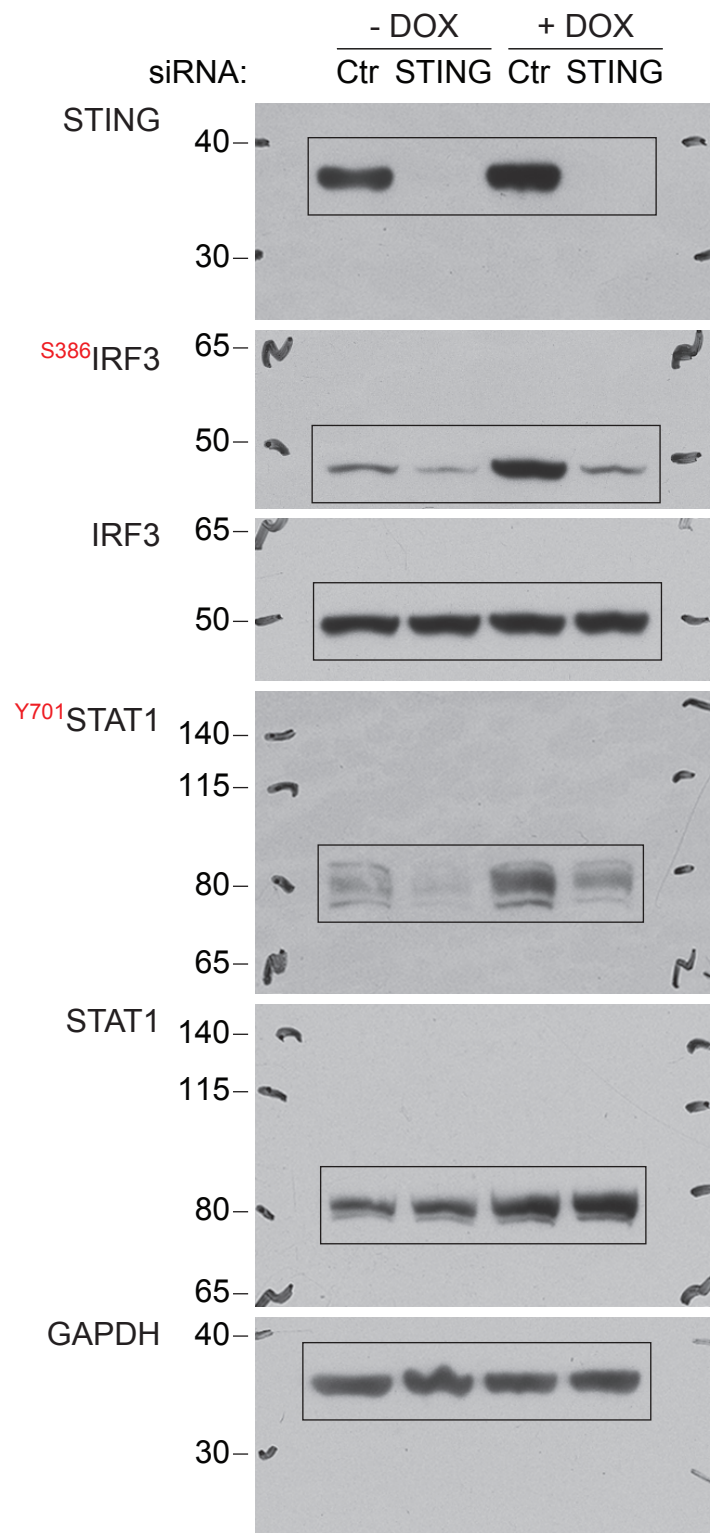

**C**

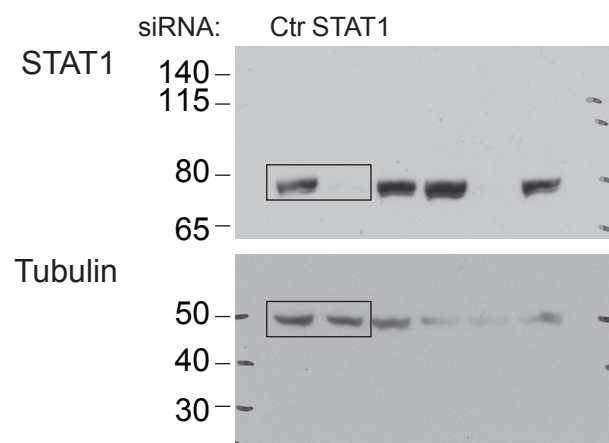

Supplement: Supplementary file 7 — Source Data [file 41467_2019_11048_MOESM7_ESM.zip › Source data 1.pdf]
